# Biarticular gastrocnemii muscles increase their joint energy transfer potential at high running speeds

**DOI:** 10.1098/rsos.241933

**Published:** 2025-04-23

**Authors:** Sebastian Bohm, Christos Theodorakis, Morteza Ghasemi, Falk Mersmann, Adamantios Arampatzis

**Affiliations:** ^1^ Department of Training and Movement Sciences, Humboldt-Universität zu Berlin, Berlin, Germany

**Keywords:** locomotion, sprinting, triceps surae muscle, energy distribution

## Abstract

The involvement of energy transfer mechanisms between the ankle and the knee joint by the biarticular gastrocnemii muscles at high running speeds is currently unknown. During running at seven speeds (3.0–8.5 m s^−1^), the ankle and knee joint kinematics as well as the electromyographic activity of the gastrocnemius medialis and lateralis were captured. By means of the ankle–knee joint coupling angles, we determined the energy transfer potential between the two joints as the fraction of contact time where the joint angles are in-phase. At speeds above 6.0 m s^−1^, the ankle-to-knee joint energy transfer potential during the first part of stance and the knee-to-ankle energy transfer potential during the second part of stance were increased by 37% and 12%, respectively. This was accompanied by a 2.8-fold and 2.0-fold increase of the gastrocnemii muscle activation. The findings demonstrate a speed-dependent modification of the ankle–knee joint coordination towards an in-phase pattern in combination with an increase in muscle activation, which enhances the possibility of energy transfer between the two joints by the biarticular gastrocnemii muscles. An increased energy transfer from the knee to the ankle joint is probably necessary to increase the power output at the ankle joint, required for the highest running speeds.

## 1. Introduction

During human running, the ankle and hip joint are important sources of mechanical power and work [1]. As running speed increases, there is a corresponding increase in power and work at the ankle joint during push off to increase stride length and at the hip joint during swing for increasing stride rate [2–5]. The required increase of joint power and work at the ankle joint can be provided by the monoarticular soleus muscle [6,7] as the largest plantar flexor [8] but also by more proximal knee and hip extensor muscles, through the function of several biarticular muscles within the lower limb. The gastrocnemii, rectus femoris and hamstring muscles, due to their biarticular nature, can transfer energy and power from the hip to the knee joint and ultimately to the ankle joint and vice versa, which allows the work and power at the joint where it is required to be increased [9–11].

For the ankle and knee joints, the energy transfer by the biarticular gastrocnemii can integrate the proximal vastus muscles, which have a greater capacity to generate work and power than the distal triceps surae muscles [12] because of their larger volume [13,14], into the absorption and generation of work and power at the ankle joint [12,15]. Arampatzis *et al*. [16] showed that at submaximal running speeds, during the first part of the stance phase, energy is transferred from the ankle to the knee joint, facilitating the energy absorption at the ankle joint. In the second part of the stance, energy is transferred from the knee to the ankle joint, enabling a contribution to the ankle joint work and power during push off, which accounts for 16% of the total ankle joint work performed by the soleus and gastrocnemii muscles together [16]. An important involvement of the biarticular energy transfer via the gastrocnemii was also found for increasing walking speeds [17] and during the recovery of body stability after drop-like and trip-like gait perturbations [18]. A recent musculoskeletal modelling study [19] further predicted that a higher energy absorption in the lower leg joints is associated with lower anterior cruciate ligament loading during landing. Taking into account the transfer of energy from the ankle to the knee joint by the gastrocnemius muscles during landing [12], this suggests that muscle biarticularity could also play an important role in managing energy dissipation and may, therefore, be relevant with regard to injury risk as well. The biarticular muscle function has also inspired robotics, and its implementation allows for bipedal robot locomotion closer to human-like dynamics, greater robustness and higher energy efficiency [20]. Together these studies demonstrate the relevance and applications of the biarticular energy transfer during motion.

It is still unclear how biarticular mechanisms might be modulated at high or maximal running speed, where essentially higher power at the ankle joint is required. In the study by Arampatzis *et al*., it was found that with increasing running speed from 2.0 to 3.5 m s^−1^, the transfer of power from the knee to the ankle joint increased by approximately 50% [16]. Some previous studies acknowledged the importance of the knee-to-ankle joint energy transfer alongside the hip-to-knee joint energy transfer during explosive movements [21–23]. For example, musculoskeletal models predicted that the contribution of the knee-to-ankle joint energy transfer to the total positive mechanical work at the ankle joint was 23–25% during maximal vertical jumps [12,24,25] and 28% during sprint push off [25]. These findings suggest a higher involvement of biarticular energy transfer mechanisms when increasing running speed from slow to maximal.

There are two conditions at which the biarticular gastrocnemii may influence the work and power at the ankle joint in addition to their own musculotendinous work and power production [11,15,20]. When the power of the gastrocnemii muscles at the knee and ankle joint has opposing signs, energy is transferred between the two joints. The same signs of the power of the gastrocnemii muscles at the knee and ankle joint reflect a simultaneous absorption or generation, i.e. distribution of their work and power at the two joints [15,16]. Considering that the gastrocnemii generate a plantar flexion and knee flexion moment, a simultaneous plantar flexion and knee extension or dorsiflexion and knee flexion (i.e. in-phase fluctuations at both joints) indicates that the power of the gastrocnemii at the two joints has opposing signs. At simultaneous knee flexion and plantar flexion or knee extension and dorsiflexion (i.e. anti-phase fluctuations at the two joints), the power of the gastrocnemii has the same sign at the two joints. Therefore, in-phase fluctuations demonstrate a possibility of an ankle-to-knee or knee-to-ankle joint energy transfer by the gastrocnemii muscles, whereas anti-phase fluctuations demonstrate a possibility of simultaneous energy production or absorption at both joints. This shows that kinematic analysis of the knee and ankle joint can provide insight into the possible involvement of biarticular mechanisms of the gastrocnemii during running.

In the current study, lower leg kinematics and electromyographic activity (EMG) of the gastrocnemius lateralis (GL) and medialis (GM) muscle were recorded during running from slow up to maximal speed. By means of vector coding, we calculated the coupling angles of the knee joint and ankle joint angles to determine in-phase and anti-phase fluctuations of the two joint angles. The energy transfer potential was then determined as the fraction of stance time where the knee joint and ankle joint angles are in-phase. The main study objective was to advance the understanding of the modulation of the energy transfer between the knee and ankle joint via the biarticular gastrocnemii muscles with increasing speed of running. This understanding has implications for the conceptualization of training and rehabilitation interventions as well as the design of exoskeletons, legged robots and assistive devices. We hypothesized that the biarticular energy transfer mechanisms become more involved at high running speed.

## 2. Methods

### 2.1. Participants and experimental gait protocol

Eighteen male participants (179 ± 7 cm, 74 ± 11 kg, 24 ± 4 years) were recruited. Inclusion criteria were (i) several years of experience in sprinting-related training, (ii) experience in treadmill running and (iii) absence of skeletal or neuromuscular impairments in the six months prior to the investigation. Participants had various sport backgrounds (e.g. basketball, soccer, track and field), but training volume, intensity and frequency were not further documented. The present study was approved by the local university ethics committee (HU-KSBF-EK_2022_0003) and conducted in accordance with the Declaration of Helsinki. Participants provided written informed consent prior to the investigation.

During a separate preparation session, the participants were familiarized with running at submaximal and maximal running speeds on a treadmill (hp/cosmos, 190/65 pulsar® 3 p). The measurement protocol included running at 3.0–8.0 m s^−1^ in 1.0 m s^−1^ increments and at maximal running speed (8.5 ± 0.3 m s^−1^). To determine the maximal running speed of each individual, the speed of the treadmill was increased from 8.0 m s^−1^ or 8.5 m s^−1^ in 0.25 m s^−1^ intervals according to the expected maximal speed obtained during the earlier familiarization session. Two to three attempts to increase the speed were made for each increment. At each of the targeted speeds, at least five steps were captured (i.e. steady state). The participants were secured during running by a safety harness, and a rest of 3–5 min between the different gait speed trials was given.

### 2.2. Joint kinematics and electromyographic activity

The kinematics of the knee and ankle joint of the right leg were recorded during running by means of a motion capture system (Vicon Motion Systems, Oxford, UK, 250 Hz) and retro-reflective markers placed on tuber calcanei, second metatarsal, lateral malleolus, lateral femoral epicondyle and greater trochanter. The three-dimensional marker coordinates were processed by a second-order low-pass filter (Butterworth) with a 6 Hz cut-off frequency. The consecutive extension maxima of the knee joint were used to define the touchdown and toe-off [26]. The knee and ankle joint angles in sagittal plane during the stance phases were time-normalized and then averaged over the five steps. For the ankle angle, 0° indicates the shank perpendicular to the foot and values <0° a dorsiflexion, while for the knee joint an angle of 180° indicates a fully extended knee and values <180° a knee joint flexion, respectively. Stance times, swing times, cadence (number of steps per second) and duty factor (portion of gait cycle with ground contact) were calculated accordingly.

A wireless EMG system (Myon m320RX, Myon AG, Baar, Switzerland, 1000 Hz) was used to measure the surface EMG of the GM and GL muscles of the right leg (same steps as kinematic analysis). Electrodes were placed according to the Seniam guidelines (https://www.seniam.org). The raw EMG data were processed with a fourth-order high-pass filter (Butterworth) with a cut-off frequency of 20 Hz, a full-wave rectification and finally a low-pass filter with a cut-off frequency of 20 Hz. For each participant, the EMG values were normalized to the highest EMG value recorded during a maximal isometric plantar flexion. For this purpose, each participant was seated in a customized chair with the knee in extended position (180°), while the foot was placed on a vertical plate, positioning the ankle angle joint at 0°. Participants wore a harness that was connected to the plate by an inflexible belt strap, adjustable in length by a ratchet. The belt was tightened to create a fixed end condition for the contraction by the counter resistance of the plate and harness. Participants were then asked to perform two maximal isometric plantar flexion contractions with 1 min rest in between, and the maximal EMG value (after processing) was used for the normalization. The EMG activity of both muscles during running was captured for 10 out of the 18 participants.

To estimate muscle activation (â) based on the measured normalized EMG activity (û), we referred to the differential equation from Zajac [27], equation (2.1):


(2.1) $$\frac{d\;â\left(t\right)}{d\;t}+\left[\frac{1}{\tau_{act}}\;\cdot \;\left(\beta +\;\left[1-\beta \right]\;û\left(t\right)\right)\right]\cdot â\left(t\right)=\left(\frac{1}{\tau_{act}}\right)\;\cdot \;û\left(t\right).$$

The activation time constant ($\tau_{act}$) and the ratio of the activation to deactivation time constant (β) specific to fast and slow twitch fibres were taken from Dick *et al*. [28], considering a fibre type distribution of 50% type 1 and 2 fibres for both gastrocnemii muscles [29,30]. An average weighted activation of the gastrocnemii muscles was then calculated according to their physiological cross-sectional area ratios (i.e. 2/3 GM and 1/3 GL [8]).

### 2.3. Assessment of biarticular mechanisms

Vector coding [31,32] was used to calculate the coupling angle (*γ*) of the knee and ankle joint angles to determine the in-phase and anti-phase movements of the two joints during the stance phase. The coupling angles were calculated for each consecutive percentage of the normalized stance phase by the arcus tangent 2, i.e. over 0°−360°. To calculate an average coupling angle ($\bar{\gamma_{i}}$) across participants for each gait speed, the horizontal ($\bar{x_{i}}$) and vertical segment angle components ($\bar{y_{i}}$) at each percentage of stance were first averaged [31,33] using equations (2.2) and (2.3):


(2.2) $$\bar{x_{i}}=\frac{1}{n}\sum_{i=1}^{n}\cos\gamma_{i}$$

(2.3) $$\bar{y_{i}}=\frac{1}{n}\sum_{i=1}^{n}\sin\gamma_{i}$$

Coupling angles of 0° ≤ γ < 90° (i.e. simultaneous plantar flexion and knee extension) and 180° ≤ γ < 270° (i.e. simultaneous dorsiflexion and knee flexion) indicate in-phase fluctuations that reflect a possibility of a knee-to-ankle and ankle-to-knee joint energy transfer by the biarticular gastrocnemii muscles, respectively [16]. Coupling angles of 90° ≤ γ < 180° (i.e. plantar flexion and simultaneous knee flexion) and 270° ≤ γ < 360° (i.e. dorsi flexion and simultaneous knee extension) indicate anti-phase fluctuations. Here, the biarticular gastrocnemii have the possibility to simultaneously absorb or produce energy in the knee and ankle joint [16].

On the basis of the calculated coupling angles during the stance phase, we further determined the fraction of the stance phase where the biarticular gastrocnemii may transfer energy from the knee to the ankle joint and vice versa (in-phase) or to simultaneously absorb or produce energy in the two joints (anti-phase). We, henceforth, define the fraction of the stance time where the ankle joint and the knee joint angles are in-phase as *energy transfer potential*. Specifically, the fraction where energy can be transferred from knee to the ankle (0° ≤ γ < 90°) was defined as *knee-to-ankle joint energy transfer potential,* and the fraction where energy can be transferred from ankle to the knee (180° ≤ γ < 270°) as *ankle-to-knee joint energy transfer potential*.

### 2.4. Statistics

To investigate the effect of running speed on the temporal and spatial gait parameters, EMG activities, activations, kinematics, coupling angles and energy transfer potentials, a linear mixed model was applied with participants treated as random effect and speed as fixed effect. A post hoc analysis with Benjamini–Hochberg correction was conducted in the case of a main effect of running speed (adjusted *p* values are reported). A linear mixed model was further used to investigate the association between the weighted activation of the gastrocnemius muscles at touchdown at the different speeds and the ankle-to-knee energy transfer potential at the different speeds and also the association between the changes of the weighted activation at touchdown across running speeds with respect to the speed of 3.0 m s^−1^ and the corresponding changes of ankle-to-knee joint energy transfer potential. As tested by the Shapiro−Wilk test, normal distribution of the normalized residuals of some parameters was not given. However, linear mixed models were proposed to be robust against violations of the assumption of normality [34]. The statistical tests were conducted by means of the software R (RStudio v. 2022.07.1, RStudio Inc., MA, USA) and the respective *emmeans* and *nlme* package. The level of significance was *α* = 0.05.

## 3. Results

**Figure 1.**
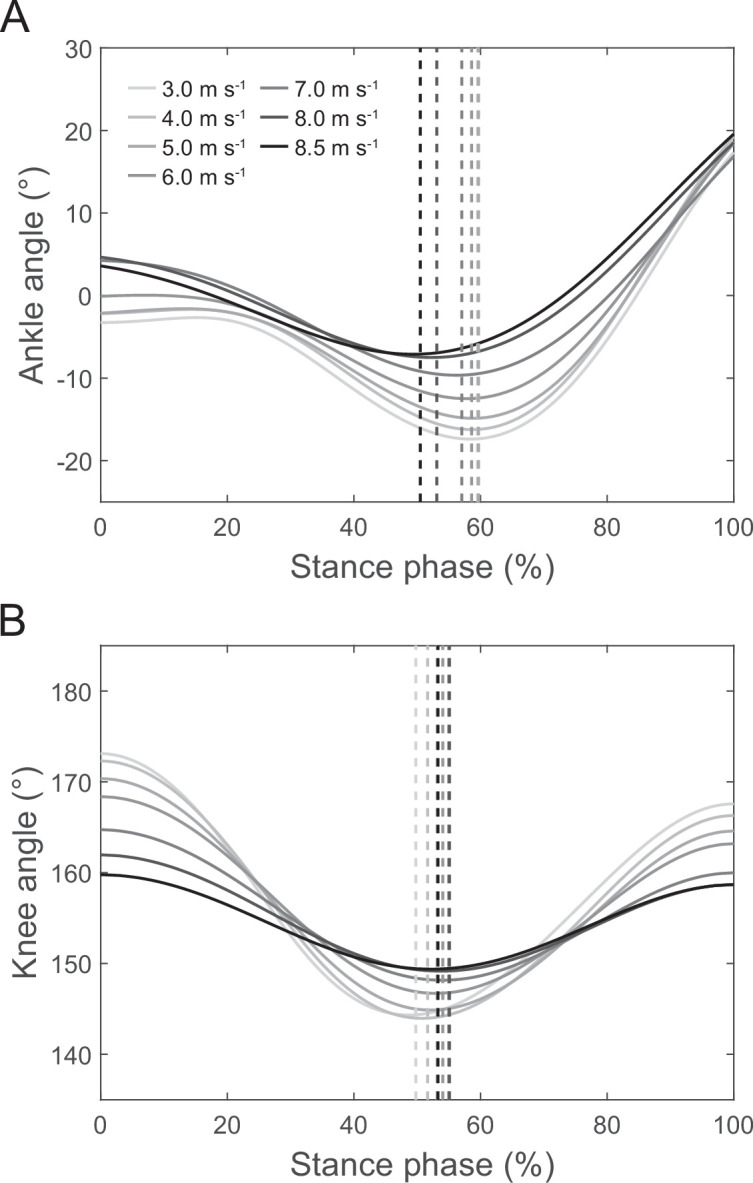
Average ankle (A) and knee joint angles (B) during the stance phase of running at the investigated speeds (*n* = 18). Dashed lines indicate instants of maximum dorsi flexion and knee flexion during the stance phase.

There was a main effect of running speed on the spatial and temporal gait parameters (*p* < 0.001). Stance and swing time as well as duty factor decreased, while cadence increased with increasing speed (table 1). Speed also showed a main effect on the ankle and knee joint kinematics (*p* < 0.001; figure 1, table 1). The ankle joint was more plantar flexed at touchdown and the knee joint more flexed at both touchdown and the end of the stance phase at higher running speeds (figure 1, table 1). With higher running speed, the maximal dorsi flexion and knee joint flexion during midstance were reduced, and the transition from dorsi to plantar flexion occurred earlier during stance, while the transition from knee flexion to extension occurred later (figure 1, table 1).

**Table 1.** Spatiotemporal and kinematic gait characteristics for the investigated running speeds (*n* = 18, mean ± s.d.)*.* TD: Touchdown; TO: Toe off.

	running speed						
	3.0 **m s^−1^**	4.0 **m s^−1^**	5.0 **m s^−1^**	6.0 **m s^−1^**	7.0 **m s^−1^**	8.0 **m s^−1^**	8.5 ± 0.3 **m s^−1^**
stance time (ms)*	302 ± 21^a^	262 ± 21 ^b^	226 ± 17^c^	196 ± 18 ^d^	164 ± 16 ^e^	142±15 ^f^	133±16 ^g^
swing time (ms)*	452 ± 28^a^	449 ± 31^a^	431 ± 29^b^	408±23 ^c^	381 ± 23 ^d^	363 ± 19 ^e^	362 ± 36 ^e^
duty factor*	0.40 ± 0.02^a^	0.37 ± 0.02^b^	0.34 ± 0.02^c^	0.33 ± 0.02 ^d^	0.30 ± 0.02 ^e^	0.28 ± 0.02^f^	0.27 ± 0.03^g^
cadence (steps/s)*	2.69 ± 0.15^a^	2.86 ± 0.16^b^	3.09 ± 0.16^c^	3.36 ± 0.17 ^d^	3.72 ± 0.19 ^e^	3.99 ± 0.26^f^	3.98 ± 0.52^f^
ankle angle TD (°)*	−3.3 ± 4.2^a^	−2.2 ± 6.5^a^	−2.1 ± 7.3^a^	−0.1 ± 8.3 ^a,b^	4.3 ± 8.5^c^	4.7 ± 7.1^c^	3.6 ± 5.2^b,c^
knee angle TD (°)*	173 ± 3^a^	172 ± 34^a^	170 ± 4^b^	168 ± 34^c^	165 ± 4^d^	163.0 ± 4^e^	160 ± 4^f^
ankle angle TO (°)	17.3 ± 5.5	19.2 ± 5.2	18.6 ± 5.3	18.4 ± 4.7	16.8 ± 4.6	18.5 ± 5.1	19.6 ± 6.2
knee angle TO (°)*	168 ± 4.6^a^	166 ± 4.7^a^	165 ± 4.6^b^	163 ± 5.3^b^	160 ± 5.4^c^	159 ± 6.2^c^	159 ± 5.7^c^
max. dorsiflexion (°)*	−17.6 ± 2.4^a^	−16.4 ± 2.4^a^	−15.1 ± 2.3^b^	−12.7 ± 2.6^c^	−9.8 ± 2.6^d^	−7.7 ± 3.3^e^	−7.4 ± 2.9^e^
max. knee flexion (°)*	144 ± 5.2^a^	144 ± 4.4^a^	145 ± 4.6^a^	146 ± 4.3^b^	148 ± 4.8^b,c^	149 ± 4.6^c^	149 ± 5.4^c^
max. dorsiflexion time (%stance)*	59.6 ± 2.8^a^	59.7 ± 2.3^a^	59.8 ± 3.0^a^	58.7 ± 3.0^a,b^	57.1 ± 3.6^b^	52.5 ± 4.1^c^	50.5 ± 4.7^d^
max. knee flexion time (%stance)*	50.0 ± 2.3^a^	51.6 ± 2.2^a,b,c^	53.1 ± 3.0^b,d^	54.1 ± 2.9^d^	55.1 ± 3.6^d^	55.2 ± 5.0^d^	53.4 ± 6.3^c,d^

^*^Significant main effect of speed (*p* < 0.05). Different letters denote significant differences between speeds (*p* < 0.05, post hoc analysis).

**Figure 2.**
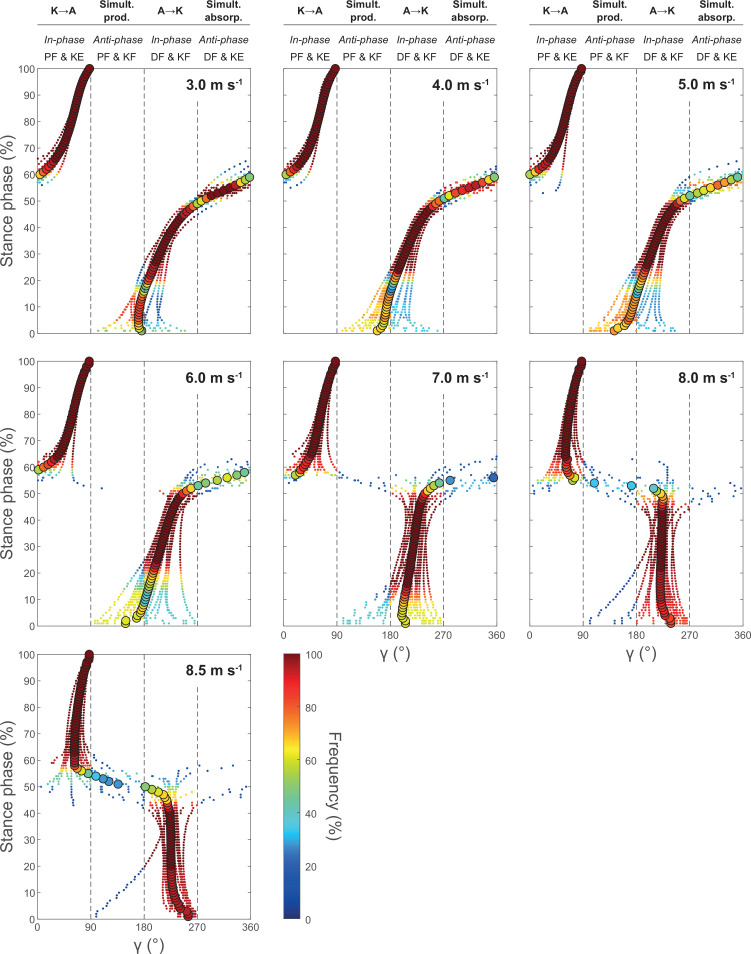
Average (large circles) and individual (small dots) coupling angles (*γ*) of the ankle and knee joint angles during the stance phase of running at the investigated running speeds. 0° < *γ* ≤ 90° indicates simultaneous plantar flexion (PF) and knee extension (KE, in-phase fluctuations) and the potential for a knee-to-ankle joint energy transfer (K→A) via the biarticular gastrocnemii muscles; 90° < *γ* ≤ 180° indicates simultaneous plantar flexion and knee flexion (KF, anti-phase fluctuations) and the potential for a simultaneous energy production at the ankle and knee joint (Simult. prod.); 180° < *γ* ≤ 270° indicates simultaneous dorsi flexion (DF) and knee flexion (in-phase fluctuations) and the potential for an ankle-to-knee joint energy transfer (A→K); 270° < *γ* ≤ 360° indicates simultaneous dorsiflexion and knee extension (anti-phase fluctuations) and the potential for simultaneous energy absorption (Simult. absorb.) at the ankle and knee joint. The colour scale represents the relative frequency of the participants in each of the four phases for every percentage of the stance phase (*n* = 18).

**Figure 3.**
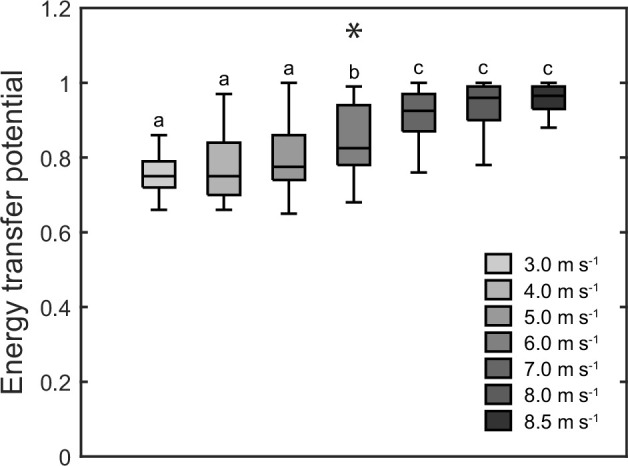
Energy transfer potential by the biarticular gastrocnemii muscles during the stance phase of running at the investigated speeds (*n* = 18). *Statistically significant main effect of speed (*p* < 0.05). Different letters denote significant differences between speeds (*p* < 0.05, post hoc analysis).

Figure 2 shows the coupling angles of the knee and ankle joint angles and the possibility of energy transfer between joints or simultaneous energy absorption/production by the biarticular gastrocnemii during the stance phase. At the submaximal speeds until 6.0 m s^−1^, there was an initial possibility of simultaneous energy production (approx. 0–15% stance), followed by a possibility of energy transfer from the ankle to the knee joint (approx. 15–50% stance), then a possibility of simultaneous energy absorption (approx. 50–60% stance) and finally a possibility of energy transfer from the knee to the ankle joint (approx. 60–100% stance). At the higher speeds above 6.0 m s^−1^, there was a clear shift from simultaneous energy production towards energy transfer from the ankle to the knee joint at the start of stance phase and from simultaneous energy absorption towards energy transfer from knee to the ankle joint during midstance (figure 2). The overall energy transfer potential during the stance phase showed a main effect of running speed (*p* < 0.001), with an overall increase of 24% (figure 3). The post hoc comparisons showed that increases were significant at 6.0 and 7.0 m s^−1^ (*p* < 0.05, figure 3).

**Figure 4.**
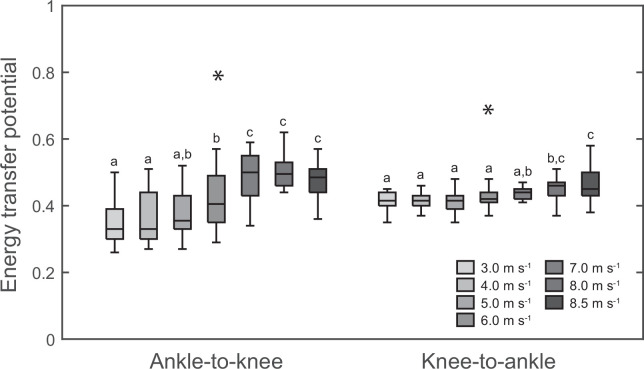
Ankle-to-knee joint and knee-to-ankle joint energy transfer potential by the biarticular gastrocnemii muscles during the stance phase of running at the investigated speeds (*n* = 18). *Statistically significant main effect of speed (*p* < 0.05). Different letters denote significant differences between speeds (*p* < 0.05, post hoc analysis).

For both the ankle-to-knee (*p* < 0.001) and the knee-to-ankle joint energy transfer potential (*p* < 0.001) there was a main effect of running speed, with an overall increase of 37% and 12%, respectively (figure 4). Post hoc comparisons indicated that the ankle-to-knee joint energy transfer potential increased significantly between 5.0 and 7.0 m s^−1^ but not before or thereafter (*p* < 0.05). While the knee-to-ankle joint energy transfer potential was not different until 7.0 m s^−1^, it was significantly higher at the two fastest speeds of 8.0 and 8.5 m s^−1^ (*p* < 0.05, figure 4).

**Figure 5.**
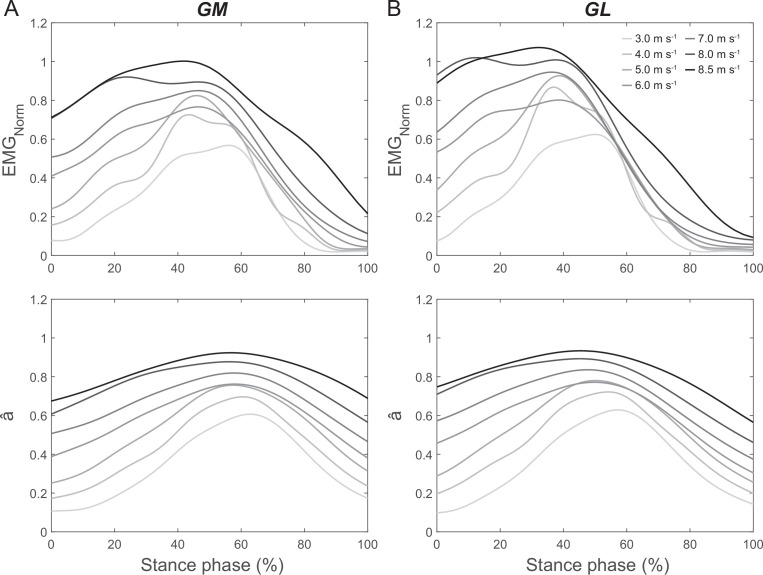
Average electromyographic activity (EMG_Norm_, normalized to a maximum voluntary plantar flexion contraction) and activation (â) of the gastrocnemius medialis (GM, column A) and gastrocnemius lateralis (GL, column B) muscles during the stance phase of running at the investigated speeds (*n* = 10).

**Figure 6.**
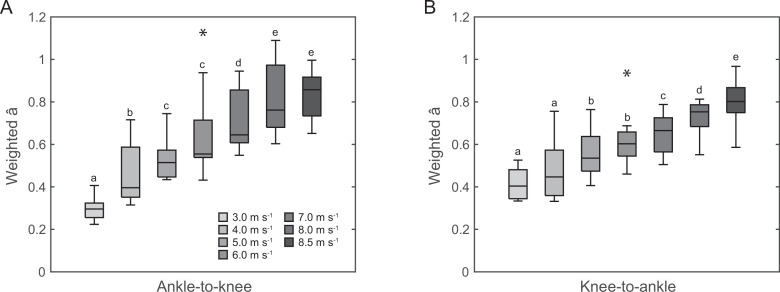
Average weighted activation (â) of the gastrocnemius medialis and gastrocnemius lateralis muscles during the phase of ankle-to-knee (A) and knee-to-ankle joint energy transfer (B) for the investigated running speeds (*n* = 10). *Statistically significant main effect of speed (*p* < 0.05). Different letters denote significant differences between speeds (*p* < 0.05, post hoc analysis).

**Figure 7.**
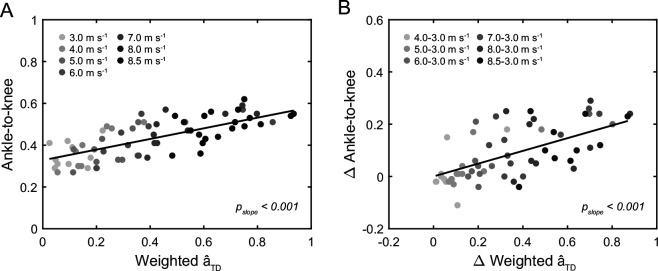
Relationship of weighted activation (â) of the gastrocnemii at touchdown (TD) and the ankle-to-knee joint energy transfer potential across running speeds (*p*_slope_< 0.001, A) as well as relationship of changes in weighted â at TD between running speeds with respect to 3.0 m s^−1^ and corresponding changes in ankle-to-knee joint energy transfer potential (*p*_slope_< 0.001, n = 10, B).

The average and maximal EMG activity and activation of the GM and GL increased continuously across speeds, all parameters showing a main effect of speed (*p* < 0.001, figure 5, table 2). There was a main effect of speed on the average weighted activation of GM and GL during the ankle-to-knee and knee-to-ankle joint energy transfer phases (*p* < 0.001, figure 6), indicating a functional relation with the energy transfer potentials. The post hoc comparison revealed continuous significant increases of the weighted activation with speed increments during the ankle-to-knee and knee-to-ankle joint energy transfer phases (*p* < 0.05, figure 6). Moreover, there was a significant effect of the weighted activation at touchdown on the ankle-to-knee joint energy transfer potential across speeds (*p*_slope_ < 0.001, figure 7). There was also a significant effect of the changes of weighted activation across the different speeds with respect to the speed of 3.0 m s^−1^ on the corresponding changes of ankle-to-knee joint energy transfer potential (*p*_slope_ < 0.001, figure 7).

**Table 2.** Electromyographic activity (EMG_Norm_, normalized to a maximal voluntary plantar flexion contraction) and activation (â) of the gastrocnemius medialis (GM) and lateralis (GL) muscles averaged over the stance phase at the investigated running speeds (*n* = 10, mean ± s.d.).

	running speed						
	3.0 **m s^−1^**	4.0 **m s^−1^**	5.0 **m s^−1^**	6.0 **m s^−1^**	7.0 **m s^−1^**	8.0 **m s^−1^**	8.5 ± 0.3 **m s^−1^**
GM EMG_Norm_*	0.27 ± 0.05^a^	0.35 ± 0.10^a,b^	0.43 ± 0.08^b,c^	0.49 ± 0.08^c,d^	0.56 ± 0.08^d^	0.67 ± 0.13^e^	0.77 ± 0.20^f^
GL EMG_Norm_*	0.28 ± 0.13^a^	0.39 ± 0.25^a,b^	0.46 ± 0.23^b,c^	0.48 ± 0.20^b,c^	0.55 ± 0.25^c,d^	0.65 ± 0.34^d,e^	0.73 ± 0.30^e^
GM â*	0.35 ± 0.05^a^	0.44 ± 0.09^b^	0.53 ± 0.08^c^	0.60 ± 0.09^d^	0.68 ± 0.07^e^	0.77 ± 0.07^f^	0.83 ± 0.08^g^
GL â*	0.36 ± 0.12^a^	0.46 ± 0.21^b^	0.55 ± 0.20^c^	0.60 ± 0.20^c^	0.68 ± 0.22^d^	0.77 ± 0.25^e^	0.82 ± 0.23^e^

^*^Significant main effect of speed (*p* < 0.05). Different letters denote significant differences between speeds (*p* < 0.05, post hoc analysis).

## 4. Discussion

We investigated the potential for energy transfer between the knee and ankle joint by the biarticular gastrocnemii muscles from slow to maximal running speed. By means of a kinematic-based approach, we found that at high running speeds above 6.0 m s^−1^, the ankle-to-knee joint energy transfer potential during the first part of the stance phase as well as the knee-to-ankle joint energy transfer potential during the second part of the stance were significantly increased. The increases of the energy transfer potentials were accompanied by significantly higher activation of the GM and GL muscles. These findings suggest a speed-dependent involvement of biarticular energy transfer between the knee and ankle joint by the gastrocnemii.

With increasing running speed from slow (3.0 m s^−1^) to maximum (8.5 ± 0.3 m s^−1^), stance time, swing time as well as duty factor decreased whereas cadence increased in magnitudes that agree with previous studies [35,36]. The observed ankle and knee joint angles as well as EMG activities during the stance phase also correspond to earlier reports [37–39]. At the submaximal speeds (3.0–6.0 m s^−1^), there was a simultaneous knee flexion and dorsiflexion at approximately 15–50% stance, indicating a possibility of an energy transfer from ankle to the knee joint via the gastrocnemii to facilitate energy absorption at the ankle joint. During approximately 60–100% stance, there was a simultaneous knee extension and plantar flexion, thus a knee-to-ankle energy transfer possibility that may enable an increase of power output at the ankle joint during push off. The overall energy transfer potential was quite high with 0.76−0.79 and accompanied by significant activation of the GL and GM muscle, indicating functionally relevant energy transfer between both joints. The importance of energy transfer has been demonstrated previously for submaximal running speeds (2.0–3.5 m s^−1^), where the transferred energy from the knee joint to the ankle joint accounted for almost half of the total musculotendinous work of the gastrocnemii muscles at the ankle joint and accordingly 16% of the ankle joint work performed by the gastrocnemii and soleus muscles together [16].

At the high and maximal speeds of 7.0−8.5 m s^−1^, the overall energy transfer potential increased from 0.76−0.79 to 0.92−0.95. This means that almost throughout the whole stance phase, there was the possibility of energy transfer between the knee and ankle joint. During the initial part of the stance, the ankle-to-knee joint energy potential increased by 37%, mainly due to a direct dorsi flexion starting at touchdown. During midstance, the transition from dorsi to plantar flexion occurred earlier, while the transition from knee joint flexion to extension occurred later. This eliminated the phase of simultaneous absorption observed during submaximal running entirely and resulted in a 12% increase of the knee-to-ankle joint energy transfer potential. The increased energy transfer potentials were accompanied by a 2.8-fold and a 2.0-fold higher muscle activation during the ankle-to-knee as well as knee-to-ankle joint energy transfer phases, respectively. Furthermore, there was a significant relation between muscle activation at touchdown in the different speeds and the ankle-to-knee joint energy transfer potential in the different speeds as well as a relation of the changes in these two parameters with speed, indicating that an increased muscle pre-activation with speed influences the kinematic pattern of the ankle and knee joints in favour of the ankle-to-knee joint energy transfer potential at the higher speeds. These findings together provide evidence for a speed-dependent change in the coordination of knee and ankle joint angles towards an in-phase pattern in combination with a simultaneous increase of muscle activation, two mechanisms that may enhance the biarticular energy transfer by the gastrocnemii at high and maximal running speeds. The speed-dependent increase of the knee-to-ankle joint energy transfer in particular would improve the required power output at the ankle joint for push off to achieve high speeds. Compared with the proximal vastus muscles, the distal triceps surae muscles are less voluminous and have shorter fascicles [13,14,40], which reduces inertial moments [9,12] and energetic cost per unit force [41], advantageous for efficient running. On the other side, the smaller volume reduces the amount of energy that can be produced by the triceps surae [12]. The knee-to-ankle energy transfer can enhance the production of energy at the ankle joint during running by involving the voluminous vastus muscles [16] that can produce more work/power.

The speed-dependent increase of the ankle-to-knee joint transfer of energy in the first part of the stance may facilitate the storage of elastic strain energy in tendinous tissues. Arampatzis *et al*. previously argued that during submaximal running, the transferred energy from the ankle joint to the knee joint in the first part of the stance phase is not dissipated but stored in the patellar–quadriceps tendon as elastic energy [16]. This is because the vastus muscles contract almost isometrically, while the vastus muscle-tendon unit is elongated due to knee flexion in the first part of the stance [42,43]. During the second part of the stance, the stored elastic strain energy is then released and transferred from the knee joint to the ankle joint via the gastrocnemii to contribute to the ankle joint energy/power production. At high running speeds, the ankle-to-knee as well as the knee-to-ankle joint energy transfer potentials and respective gastrocnemii muscle activation were substantially increased, indicating an enhanced energy transfer between joints that finally may have contributed to achieving the high required power at the ankle joint. Considering a higher EMG activity of the vastus muscles in the first part of the stance [44], the energy transfer between both joints may theoretically also involve increased storage and recoil of elastic strain energy in and from the patellar–quadriceps tendon as described above. The relevance of the knee-to-ankle energy transfer for the ankle joint push off has previously been suggested for other explosive leg extension movements [21–25] and is in agreement with the implications from the present study. Furthermore, an increased knee-to-ankle joint energy transfer may partly compensate for limitations of the force-generating capacity of the plantar flexors at high speeds [15]. In fact, a critical contractile condition for force generation at high running speeds has been shown for the monoarticular soleus as the main plantar flexor muscle, with a reduction in the fascicles’ force–velocity potential from 0.53 to 0.28 from slow to maximal speed [45]. Note that the biarticular energy transfer within the lower limbs is not limited to the ankle and knee joint. Via the biarticular rectus femoris and hamstrings, work and power can also be transferred from the hip to the knee joint and ultimately to the ankle joint and vice versa. During running, a proximal to distal energy transfer in particular may contribute to the required power and work at the ankle joint [12,25].

The knee-to-ankle and ankle-to-knee joint energy transfer potentials only indicate the possibility of energy transfer between both joints and not the magnitude. For the quantification of the magnitude of energy transfer, the investigation of the power of the gastrocnemii muscles at both joints is required [15–17]. The identified fractions of possible energy transfer during stance at the slow speeds in the present study agree quite well with our experimental findings of a relevant power of the gastrocnemii at the knee and ankle joints [16]. This shows that through recordings of kinematics and EMG activities of the gastrocnemii muscles, the investigation of the involvement of biarticular mechanisms during running is possible. In the present study, participants were investigated during running on a motorized treadmill, which might be limited regarding its ecological validity. However, a recent review with meta-analysis on 33 studies (494 participants) concluded that spatiotemporal, kinematic, kinetic, muscle activity and muscle–tendon outcome measures are largely comparable between treadmill and overground running [46]. Therefore, though some slight differences between overground and treadmill cannot be ruled out, their effect should be small and not affect the outcome of the current study. We used a kinematic-based approach to detect the touchdown and toe off during running. Inaccuracies of the touchdown detection could theoretically affect the observed speed-related increases in the ankle-to-knee energy transfer potential because they were mainly caused by a direct dorsi flexion starting at touchdown at the higher speeds. However, when performing a sensitivity analysis by substantially postponing the touchdown by 20 ms (15% of the stance phase in the highest speed), the increase in the ankle-to-knee joint energy transfer potential remained significant (main effect of speed *p* < 0.001, post hoc: 3.0 m s^−1^ 0.35 ± 0.53^a^, 4.0 m s^−1^ 0.37 ± 0.64 ^a,b^, 5.0 m s^−1^ 0.39 ± 0.56 ^b,c^, 6.0 m s^−1^ 0.42 ± 0.54 ^c,d^, 7.0 m s^−1^ 0.46 ± 0.40^e^, 8.0 m s^−1^ 0.42 ± 0.56^d^, 8.5 m s^−1^ 0.39 ± 0.58 ^b,d^, different letters denote significant differences between speeds *p* < 0.05). This indicates that the results of the present study are quite robust against touchdown inaccuracies. Finally, we estimated muscle activation because it is currently not possible to measure activation *in vivo*. For this purpose, we used the differential equation by Zajac [27], which is established in the field. We further adjusted the parameter for the activation time constant for the slow and fast twitch fibre type distribution [28] of the GM and GL (50/50%) [29,30] to improve validity.

In conclusion, our results showed a clear speed-dependent modification of the coordination of knee and ankle joint angles towards in-phase fluctuations in combination with an increase of the gastrocnemii muscle activation, which enhanced the possibility of an ankle-to-knee and knee-to-ankle energy transfer by the biarticular gastrocnemii. An increased involvement of biarticular energy transfer between the two joints is of high functional relevance because it enables a higher contribution of power from the knee at the ankle joint, required to achieve maximal running speeds. Furthermore, the enhanced biarticular energy transfer between joints seems to integrate a considerable strain energy storing and recoil by the elastic elements of the vastus muscle-tendon units. These insights into the interplay between muscle activation and biarticular mechanisms responsible for the energy transfer between the lower leg joints have implications for the conceptualization of training and rehabilitation interventions in sport, clinical and geriatric contexts as well as the design of exoskeletons, legged robots and assistive devices.

## Data Availability

The dataset can be accessed online: https://doi.org/10.6084/m9.figshare.28485476.v1.

## References

[1] Winter DA. 1983 Moments of force and mechanical power in jogging. J. Biomech. **16**, 1. (10.1016/0021-9290(83)90050-7) 6833314

[2] Arampatzis A, Brüggemann GP, Metzler V. 1999 The effect of speed on leg stiffness and joint kinetics in human running. J. Biomech. **32**, 1349–1353. (10.1016/s0021-9290(99)00133-5) 10569714

[3] Belli A, Kyröläinen H, Komi PV. 2002 Moment and power of lower limb joints in running. Int. J. Sports Med. **23**, 136–141. (10.1055/s-2002-20136) 11842362

[4] Schache AG, Brown NAT, Pandy MG. 2015 Modulation of work and power by the human lower-limb joints with increasing steady-state locomotion speed. J. Exp. Biol. **218**, 15. (10.1242/jeb.119156) 26056240

[5] Willer J, Allen SJ, Burden RJ, Folland JP. 2024 How humans run faster: the neuromechanical contributions of functional muscle groups to running at different speeds. Scand. J. Med. Sci. Sports **34**, e14690. (10.1111/sms.14690) 39049546

[6] Lai A, Schache AG, Lin YC, Pandy MG. 2014 Tendon elastic strain energy in the human ankle plantar-flexors and its role with increased running speed. J. Exp. Biol. **217**, 17. (10.1242/jeb.100826) 24948642

[7] Swinnen W, Mylle I, Hoogkamer W, De Groote F, Vanwanseele B. 2022 Triceps surae muscle force potential and force demand shift with altering stride frequency in running. Scand. J. Med. Sci. Sports **32**, 1444–1455. (10.1111/sms.14209) 35839378

[8] Albracht K, Arampatzis A, Baltzopoulos V. 2008 Assessment of muscle volume and physiological cross-sectional area of the human triceps surae muscle in vivo. J. Biomech. **41**, 2211–2218. (10.1016/j.jbiomech.2008.04.020) 18555257

[9] Cleland J. 1867 On the actions of muscles passing over more than one joint. J. Anat. Physiol. **1**, 85–93. PMC1318532 17230710

[10] Fick AE. 1879 Über zweigelenkige Muskeln. Archiv Für Anatomie Und Physiologie Anatomische Abteilung **3**, 201–239.

[11] van Ingen Schenau GV. 1989 From rotation to translation: constraints on multi-joint movements and the unique action of bi-articular muscles. Hum. Mov. Sci **8**, 301–337. (10.1016/0167-9457(89)90037-7)

[12] Prilutsky BI, Zatsiorsky VM. 1994 Tendon action of two-joint muscles: transfer of mechanical energy between joints during jumping, landing, and running. J. Biomech. **27**, 25–34. (10.1016/0021-9290(94)90029-9) 8106533

[13] Mersmann F, Bohm S, Schroll A, Boeth H, Duda G, Arampatzis A. 2015 Muscle shape consistency and muscle volume prediction of thigh muscles. Scand. J. Med. Sci. Sports **25**, e208–13. (10.1111/sms.12285) 24975992

[14] Mersmann F, Bohm S, Schroll A, Arampatzis A. 2014 Validation of a simplified method for muscle volume assessment. J. Biomech. **47**, 1348–1352. (10.1016/j.jbiomech.2014.02.007) 24607005

[15] Prilutsky BI, Herzog W, Leonard T. 1996 Transfer of mechanical energy between ankle and knee joints by gastrocnemius and plantaris muscles during cat locomotion. J. Biomech. **29**, 391–403. (10.1016/0021-9290(95)00054-2) 8964769

[16] Arampatzis A, Kharazi M, Theodorakis C, Mersmann F, Bohm S. 2023 Biarticular mechanisms of the gastrocnemii muscles enhance ankle mechanical power and work during running. R. Soc. Open Sci. **10**, 230007. (10.1098/rsos.230007) 37650058 PMC10465202

[17] Kharazi M, Theodorakis C, Mersmann F, Bohm S, Arampatzis A. 2023 Contractile work of the soleus and biarticular mechanisms of the gastrocnemii muscles increase the net ankle mechanical work at high walking speeds. Biology **12**, 872. (10.3390/biology12060872) 37372156 PMC10295290

[18] Theodorakis C, Bohm S, Epro G, Mersmann F, Werth J, Karamanidis K, Arampatzis A. 2025 Enhanced joint energy transfer potential by the biarticular gastrocnemii muscles during perturbed walking. Eur. J. Appl. Physiol. (10.1007/s00421-025-05727-z) PMC12354497 40042657

[19] Xu D, Zhou H, Quan W, Gusztav F, Wang M, Baker JS, Gu Y. 2023 Accurately and effectively predict the ACL force: Utilizing biomechanical landing pattern before and after-fatigue. Comput. Methods Programs Biomed. **241**, 107761. (10.1016/j.cmpb.2023.107761) 37579552

[20] Junius K, Moltedo M, Cherelle P, Rodriguez-Guerrero C, Vanderborght B, Lefeber D. 2017 Biarticular elements as a contributor to energy efficiency: biomechanical review and application in bio-inspired robotics. Bioinspir. Biomim. **12**, 061001. (10.1088/1748-3190/aa806e) 28718780

[21] Gregoire L, Veeger HE, Huijing PA, van Ingen Schenau GJ. 1984 Role of mono- and biarticular muscles in explosive movements. Int. J. Sports Med. **05**, 301–305. (10.1055/s-2008-1025921) 6511147

[22] Jacobs R, van Ingen Schenau GJ. 1992 Intermuscular coordination in a sprint push-off. J. Biomech. **25**, 953–965. (10.1016/0021-9290(92)90031-u) 1517272

[23] van Ingen Schenau GJ, Bobbert MF, Rozendal RH. 1987 The unique action of bi-articular muscles in complex movements. J. Anat. **155**, 1–5. 3503041 PMC1261869

[24] Bobbert MF, Huijing PA, van Ingen Schenau GJ. 1986 An estimation of power output and work done by the human triceps surae musle-tendon complex in jumping. J. Biomech. **19**, 899–906. (10.1016/0021-9290(86)90185-5) 3793738

[25] Jacobs R, Bobbert MF, van Ingen Schenau GJ. 1996 Mechanical output from individual muscles during explosive leg extensions: the role of biarticular muscles. J. Biomech. **29**, 513–523. (10.1016/0021-9290(95)00067-4) 8964781

[26] Fellin RE, Rose WC, Royer TD, Davis IS. 2010 Comparison of methods for kinematic identification of footstrike and toe-off during overground and treadmill running. J. Sci. Med. Sport **13**, 646–650. (10.1016/j.jsams.2010.03.006) 20478742 PMC3266867

[27] Zajac FE. 1989 Muscle and tendon: properties, models, scaling, and application to biomechanics and motor control. Crit. Rev. Biomed. Eng. **17**, 359–411. 2676342

[28] Dick TJM, Biewener AA, Wakeling JM. 2017 Comparison of human gastrocnemius forces predicted by hill-type muscle models and estimated from ultrasound images. J. Exp. Biol. **220**, 9. (10.1242/jeb.154807) PMC5450802 28202584

[29] Edgerton VR, Smith JL, Simpson DR. 1975 Muscle fibre type populations of human leg muscles. Histochem. J. **7**, 259–266. (10.1007/bf01003594) 123895

[30] Johnson MA, Polgar J, Weightman D, Appleton D. 1973 Data on the distribution of fibre types in thirty-six human muscles. An autopsy study. J. Neurol. Sci. **18**, 111–129. (10.1016/0022-510x(73)90023-3) 4120482

[31] Hamill J, Haddad JM, McDermott WJ. 2000 Issues in quantifying variability from a dynamical systems perspective. J. Appl. Biomech. **16**, 407–418. (10.1123/jab.16.4.407)

[32] Sparrow WA, Donovan E, van Emmerik R, Barry EB. 1987 Using relative motion plots to measure changes in intra-limb and inter-limb coordination. J. Mot. Behav. **19**, 1. (10.1080/00222895.1987.10735403) 23944916

[33] Needham R, Naemi R, Chockalingam N. 2014 Quantifying lumbar–pelvis coordination during gait using a modified vector coding technique. J. Biomech. **47**, 1020–1026. (10.1016/j.jbiomech.2013.12.032) 24485511

[34] Jacqmin-Gadda H, Sibillot S, Proust C, Molina JM, Thiébaut R. 2007 Robustness of the linear mixed model to misspecified error distribution. Comput. Stat. Data Anal. **51**, 5142–5154. (10.1016/j.csda.2006.05.021)

[35] Dorn TW, Schache AG, Pandy MG. Muscular strategy shift in human running: dependence of running speed on hip and ankle muscle performance. J Exp Biol **215**, 1944–1956. 10.1242/jeb.064527 22573774

[36] Santuz A, Ekizos A, Kunimasa Y, Kijima K, Ishikawa M, Arampatzis A. Lower complexity of motor primitives ensures robust control of high-speed human locomotion. Heliyon **6**, e05377. https://www.cell.com/heliyon/abstract/S2405-8440(20)32220-9 10.1016/j.heliyon.2020.e05377 PMC7610320 33163662

[37] Kuitunen S, Komi PV, Kyröläinen H. 2002 Knee and ankle joint stiffness in sprint running. Med. Sci. Sports Exerc. **34**, 166–173. (10.1097/00005768-200201000-00025) 11782663

[38] Lai A, Lichtwark GA, Schache AG, Lin YC, Brown NAT, Pandy MG. 2015 In vivo behavior of the human soleus muscle with increasing walking and running speeds. J. Appl. Physiol. **118**, 1266–1275. (10.1152/japplphysiol.00128.2015) 25814636

[39] Novacheck TF. 1998 The biomechanics of running. Gait Posture **7**, 77–95. (10.1016/s0966-6362(97)00038-6) 10200378

[40] Ward SR, Eng CM, Smallwood LH, Lieber RL. 2009 Are current measurements of lower extremity muscle architecture accurate? Clin. Orthop. Relat. Res. **467**, 1074–1082. (10.1007/s11999-008-0594-8) 18972175 PMC2650051

[41] Biewener AA, Roberts TJ. 2000 Muscle and tendon contributions to force, work, and elastic energy savings: a comparative perspective. Exerc. Sport Sci. Rev **28**, 99–107. https://journals.lww.com/acsm-essr/abstract/2000/28030/muscle_and_tendon_contributions_to_force,_work,.2.aspx 10916700

[42] Bohm S, Marzilger R, Mersmann F, Santuz A, Arampatzis A. 2018 Operating length and velocity of human vastus lateralis muscle during walking and running. Sci. Rep. **8**, 5066. (10.1038/s41598-018-23376-5) 29567999 PMC5864755

[43] Monte A, Baltzopoulos V, Maganaris CN, Zamparo P. 2020 Gastrocnemius medialis and vastus lateralis in vivo muscle‐tendon behavior during running at increasing speeds. Scand. J. Med. Sci. Sports **30**, 1163–1176. (10.1111/sms.13662) 32227378

[44] Mero A, Komi PV. 1986 Force-, EMG-, and elasticity-velocity relationships at submaximal, maximal and supramaximal running speeds in sprinters. Eur. J. Appl. Physiol. Occup. Physiol. **55**, 553–561. (10.1007/bf00421652) 3769912

[45] Bohm S, Mersmann F, Schroll A, Arampatzis A. 2023 Speed-specific optimal contractile conditions of the human soleus muscle from slow to maximum running speed. J. Exp. Biol. **226**, 246437. (10.1242/jeb.246437) 37901934

[46] Van Hooren B, Fuller JT, Buckley JD, Miller JR, Sewell K, Rao G, Barton C, Bishop C, Willy RW. 2020 Is Motorized treadmill running biomechanically comparable to overground running? A systematic review and meta-analysis of cross-over studies. Sports Med. **50**, 785–813. (10.1007/s40279-019-01237-z) 31802395 PMC7069922

